# Retraction: Potential synergistic activity of quercetin with antibiotics against multidrug-resistant clinical strains of *Pseudomonas aeruginosa*

**DOI:** 10.1371/journal.pone.0356579

**Published:** 2026-09-08

**Authors:** The PLOS One Editors

Following the publication of this article [1] concerns were raised about results presented in Figs 6 and S4, as well as the reported statistical methodology. Specifically,

- The following panels appear similar despite being used to represent different experimental conditions:
  - Fig 6 YU-V10 Quercetin + Amikacin and Fig 6 YU-V28 Quercetin + Tobramycin
  - Fig S4 YU-V11 Control and Fig S4 YU-V15 Control
  - Fig S4 PAO1 Quercetin and Fig S4 YU-V15 Quercetin
  - Fig S4 YU-V10 Quercetin and Fig S4 YU-V11 Quercetin
- The following panels appear to partially overlap despite being used to represent different experimental conditions:
  - Fig 6 YU-V15 Quercetin + Amikacin of [1] and Fig 4 PAO1 Quercetin + Ceftriaxone of [2]
  - Fig 6 YU-V28 Quercetin + Amikacin of [1] and Fig 4 PAO1 Quercetin + Levofloxacin of [2]
- The Statistical analysis subsection of the Materials and methods section only reports the use of Student’s t-test and does not explain how the analysis was adjusted for multivariate comparisons or corrected for multiple pairwise comparisons.

Regarding the partial and full panel overlap concerns, the corresponding author stated that the panel overlaps were due to figure assembly errors. They provided duplicate underlying image data for Fig 6 and triplicate underlying image data for Fig S4. The underlying data was assessed by the PLOS editorial team and two independent members of the *PLOS One* Editorial Board, who raised additional concerns about the integrity and reliability of these data.

Regarding the statistical methodology concerns, the corresponding author stated that multiple comparisons among different antibiotics or between different strains were not conducted. They commented that the objective was specifically to determine the extent to which cell killing was reduced by each drug combination relative to the infected control group, and clarified that the uninfected control group was included solely to assess baseline cell viability and overall cell survival. PLOS remains concerned about the methodological approach used for the statistical analysis reported in this study.

In light of the above concerns, which call into question the integrity and reliability of the published results, the *PLOS One* Editors retract this article.

PDR agreed with the retraction. CV, KS, FF, MM, SSR, A, and ABA either did not respond directly or could not be reached.
